# Phenotypic Resistance Profiles, Biofilm Formation, and In Vitro Carbapenem-Sparing Antimicrobial Activity in Enterobacterales Causing Acute Pyelonephritis

**DOI:** 10.3390/microorganisms14061287

**Published:** 2026-06-06

**Authors:** Livia Stanga, Ovidiu Rosca, Iulia Georgiana Bogdan, Ciprian Ilie Roșca, Horia Silviu Branea, Camelia Vidița Gurban

**Affiliations:** 1 Discipline of Microbiology, Faculty of Medicine, “Victor Babeș” University of Medicine and Pharmacy, 300041 Timișoara, Romania; stanga.livia@umft.ro; 2 Department of Infectious Diseases, “Victor Babeș” University of Medicine and Pharmacy, 300041 Timișoara, Romania; rosca.ovidiu@umft.ro (O.R.); bogdan.iulia@umft.ro (I.G.B.); 3 Department V—Internal Medicine I, “Victor Babeș” University of Medicine and Pharmacy, 300041 Timișoara, Romania; 4 Department of Internal Medicine I—Medical Semiotics II, “Victor Babeș” University of Medicine and Pharmacy, 300041 Timișoara, Romania; 5 Department IV—Biochemistry and Pharmacology, “Victor Babeș” University of Medicine and Pharmacy, 300041 Timișoara, Romania; gurban.camelia@umft.ro

**Keywords:** pyelonephritis, β-lactamases, biofilms, microbial sensitivity tests, enterobacteriaceae infections

## Abstract

Empirical management of acute pyelonephritis in Eastern Europe is increasingly constrained by extended-spectrum β-lactamase (ESBL)-producing Enterobacterales and by uropathogen phenotypes—such as strong biofilm formation—which may further blunt antimicrobial activity. We aimed to characterise resistance mechanisms, minimum inhibitory concentration (MIC) distributions, biofilm-forming capacity, and the in vitro performance of carbapenem-sparing agents and to test whether these microbiological features improve prediction of clinical failure beyond standard bedside risk scores. We retrospectively analysed 102 Enterobacterales isolates recovered from 129 consecutive culture-confirmed adult pyelonephritis admissions at “Victor Babeș” University Hospital, Timișoara (March 2022–March 2025). MIC values were determined by Vitek 2 and interpreted using EUCAST v13 breakpoints; ESBL, AmpC, and carbapenemase phenotypes were confirmed by combination disk and modified carbapenem inactivation methods. Biofilm formation was quantified by the microtiter-plate crystal-violet assay. Mediation, Restricted Mean Survival Time (RMST), and decision-curve analyses were used to assess added clinical value. ESBL was confirmed in 30/102 (29.4%) isolates, AmpC in 9 (8.8%), and carbapenemase in 4 (3.9%). ESBL+ isolates were more often strong biofilm formers (33.3% vs. 12.5%; *p* = 0.014) and showed a 4- to 16-fold rightward MIC shift for cefepime, piperacillin–tazobactam, and ciprofloxacin. Among carbapenem-sparing agents, ceftazidime–avibactam (96.7% S), fosfomycin (80.0% S), and amikacin (73.3% S) retained the highest activity against ESBL+ isolates. Strong biofilm formation and the ESBL phenotype were independently associated with worse outcomes (adjusted OR 3.5 and 4.7); an exploratory mediation analysis suggested that biofilm formation may explain part of the observed association between the ESBL phenotype and treatment failure and that delayed effective therapy may account for a further portion of this association. A microbiology-enhanced model that added the ESBL phenotype, biofilm strength, and acquisition setting to routine clinical variables improved discrimination over a clinical-only baseline (AUC 0.89 vs. 0.71) and showed a higher net benefit on exploratory decision-curve analysis across the 10–40% threshold range. These predictive findings derive from a single-centre cohort with a small number of events and were only internally validated; they require validation in independent cohorts before any clinical application can be considered. The ESBL phenotype and strong biofilm formation were each independently associated with worse outcomes in pyelonephritis and may help identify candidate isolates for carbapenem-sparing strategies anchored on ceftazidime–avibactam, fosfomycin, and amikacin; given the observational, single-centre design, these associations should be regarded as hypothesis-generating.

## 1. Introduction

Acute pyelonephritis is among the most frequent infectious admissions to internal medicine and infectious-disease wards across Europe and consumes a disproportionate share of parenteral antimicrobial use [1,2]. Although community-onset pyelonephritis in otherwise healthy women has historically been treatable with narrow-spectrum agents, the steady rise of ESBL-producing Enterobacterales, AmpC-derepressed strains, and—more recently—carbapenemase-producing isolates has eroded the predictability of empirical regimens [2,3]. European Centre for Disease Prevention and Control (ECDC) surveillance consistently places Romania in the high-resistance tier for third-generation cephalosporins, fluoroquinolones, and aminoglycosides among Escherichia coli and Klebsiella pneumoniae, with tertiary-care isolates exhibiting the most complex co-resistance patterns [3,4].

The clinical translation of resistance phenotype data depends on more than dichotomous susceptible/resistant calls. The shape of the MIC distribution, the position of clinical breakpoints relative to the wild-type and non-wild-type populations, and the presence of low-level “MIC creep” all influence the probability of treatment success at standard dosing schedules [5,6]. A growing body of literature emphasises the value of inspecting full MIC histograms, particularly for carbapenem-sparing agents such as piperacillin–tazobactam, cefepime, and the novel β-lactam/β-lactamase inhibitor combinations, where isolates classified as susceptible may still cluster close to the breakpoint and behave differently in vivo [6,7]. This perspective is rarely incorporated into single-centre Romanian reports of uropathogens.

Beyond enzymatic resistance, the architecture of bacterial communities inside the urinary tract modulates treatment response. Biofilm formation by Enterobacterales—assessed phenotypically by the microtiter-plate crystal-violet method—has been linked to recurrent urinary tract infection, indwelling catheter colonisation, and persistent infection despite in vitro susceptibility of planktonic cells [8,9]. The intersection of biofilm phenotype and enzymatic resistance is less well described: recurrent antibiotic exposure may enrich biofilm-tolerant subpopulations, and ESBL-producing isolates could co-segregate with stronger biofilm formation [9,10]. Because the present study did not include molecular or genomic analysis, any common regulatory basis for these phenotypes remains speculative and cannot be addressed by our data. We therefore frame this potential co-occurrence as a hypothesis to be tested, rather than an established mechanism. If borne out, such a dual phenotype might explain part of the residual outcome gap that remains after correction for time to effective therapy.

Carbapenem-sparing antimicrobial strategies have become a stewardship priority across European Society of Clinical Microbiology and Infectious Diseases (ESCMID) guidance documents, particularly in high-resistance countries where carbapenems are the de facto empirical backbone in severely ill patients [11,12,13,14,15,16,17,18]. Candidate agents include piperacillin–tazobactam, ceftolozane–tazobactam, ceftazidime–avibactam, fosfomycin (intravenous), amikacin, and selected step-down options such as oral fosfomycin or nitrofurantoin for non-bacteraemic infection [12,13,19,20,21,22]. The relative contribution of each of these agents to a rational empirical pathway in Eastern European pyelonephritis—where ESBL prevalence among community isolates already exceeds 20%—has not been systematically benchmarked.

The interface between microbiology and bedside decision is best explored by quantitative methods that go beyond univariable association. Mediation analysis allows the total effect of a resistance phenotype on clinical failure to be partitioned into direct and indirect effects, identifying actionable points in the causal pathway [14]. Restricted Mean Survival Time (RMST) provides a clinically interpretable summary of time-to-recovery differences that does not rely on proportional hazards [15,23]. Decision-curve analysis quantifies whether the addition of microbiological features to a clinical prediction model translates into net clinical benefit at thresholds that matter for empirical-therapy decisions [16]. These techniques are seldom applied jointly in single-centre uropathogen studies.

Against this background, the present study had four objectives: first, to describe the resistance-mechanism landscape (ESBL, AmpC, and carbapenemase) and full MIC distributions of the 102 Enterobacterales isolates recovered from adult pyelonephritis at our tertiary centre; second, to characterise biofilm-forming capacity and its association with enzymatic resistance phenotype, host factors, and clinical outcome; third, to benchmark the in vitro activity of carbapenem-sparing agents and to construct a conditional-susceptibility (observational conditional probability) matrix for empirical regimen selection; fourth, to explore—using mediation, RMST, and decision-curve analyses—whether a microbiology-enhanced prediction model adds discrimination over a clinical-only baseline. Given the modest sample size and single-centre design, these latter analyses are intended as exploratory and hypothesis-generating rather than confirmatory.

## 2. Materials and Methods

### 2.1. Study Design, Setting, and Isolate Provenance

We conducted a single-centre, retrospective microbiological cohort study at “Victor Babeș” Clinical Hospital for Infectious Diseases and Pneumology, Timișoara, Romania. The hospital serves the Banat region (catchment ≈ 1.9 million inhabitants) and operates as the tertiary referral centre for adult infectious-disease admissions in Western Romania. The microbiology laboratory is accredited under ISO 15189 and participates in the national external quality assessment scheme for clinical microbiology. The study period was 1 March 2022 to 1 March 2025, covering three full post-pandemic years that follow the implementation of an updated institutional antibiogram and the introduction of ceftolozane–tazobactam and ceftazidime–avibactam into the local formulary.

All consecutive adult (≥18 years) admissions with a discharge diagnosis of acute pyelonephritis and a urinary culture growing a single uropathogen at ≥10^5^ colony-forming units (CFU)/mL (or ≥10^4^ CFU/mL with concordant blood culture) were screened. From the 129 patients meeting the parent cohort’s clinical criteria, 102 Enterobacterales isolates were retained for the present microbiology-focused analysis; the remaining 27 cases were excluded from the primary microbiological comparisons because they were caused by Pseudomonas aeruginosa (n = 11), Enterococcus spp. (n = 11), or other non-Enterobacterales organisms (n = 5). Inclusion criteria for the microbiological cohort were therefore: (i) age ≥ 18 years; (ii) a discharge diagnosis of acute pyelonephritis; (iii) a monomicrobial urine culture meeting the quantitative thresholds above; and (iv) recovery of an Enterobacterales uropathogen with a viable stored isolate available for biofilm and MIC confirmation. Exclusion criteria were: (i) polymicrobial cultures; (ii) non-Enterobacterales uropathogens (Pseudomonas aeruginosa, Enterococcus spp., and other organisms, as detailed above); (iii) cultures not meeting the quantitative significance thresholds; and (iv) absence of a recoverable stored isolate. Only one isolate per patient (the index admission isolate) was included, so the 102 isolates correspond to 102 distinct patients. All Enterobacterales isolates were prospectively stored at −80 °C in 20% glycerol broth and were thawed once for the supplementary biofilm and MIC confirmation steps described below. This study was approved by the local research ethics committee (approval 50/02.10.2023).

### 2.2. Identification, MIC Determination, and Resistance-Mechanism Confirmation

Species identification was performed by matrix-assisted laser desorption/ionisation time-of-flight mass spectrometry (MALDI-TOF MS; Bruker Biotyper, Bruker Daltonik, Bremen, Germany) on isolated colonies grown on Columbia blood agar. MIC values for 18 antimicrobial agents (ampicillin, amoxicillin–clavulanate, piperacillin–tazobactam, cefuroxime, ceftriaxone, ceftazidime, cefepime, ceftolozane–tazobactam, ceftazidime–avibactam, ertapenem, imipenem, meropenem, gentamicin, amikacin, ciprofloxacin, trimethoprim–sulfamethoxazole, fosfomycin, and nitrofurantoin) were determined using the Vitek 2 system (bioMérieux, Marcy l’Étoile, France) with AST-N400 and AST-XN17 cards, supplemented by gradient strip MIC testing (Etest, bioMérieux, Paris, France) for isolates with off-scale or borderline values. Results were interpreted using EUCAST clinical breakpoints v13.0 (2023), applied prospectively to every isolate at the time of culture report.

Phenotypic confirmation of resistance mechanisms followed EUCAST guidance. ESBL production was confirmed by the combination disk method using cefotaxime and ceftazidime, with and without clavulanic acid; a ≥5 mm increase in inhibition zone diameter in the presence of clavulanate was considered positive. Plasmid-mediated AmpC over-production was screened by reduced cefoxitin susceptibility and confirmed by the cloxacillin synergy disk test. Carbapenemase production was screened according to the EUCAST screening cut-off for meropenem (>0.25 mg/L) and confirmed by the modified carbapenem inactivation method (mCIM) with EDTA supplementation (eCIM) for metallo-β-lactamase discrimination. Multidrug resistance (MDR) was defined per the 2012 ECDC/CDC consensus as non-susceptibility to ≥1 agent in ≥3 antimicrobial categories. Quality control was performed weekly with *E. coli* ATCC 25922, *K. pneumoniae* ATCC 700603, and *P. aeruginosa* ATCC 27853. All resistance mechanisms were assigned by phenotypic methods only; no molecular or genotypic testing (e.g., PCR for bla genes or whole-genome sequencing) was performed. Accordingly, designations of ESBL, AmpC, and carbapenemase production represent phenotypic inferences rather than genotypically confirmed mechanisms. In particular, the carbapenemase phenotype could not be assigned to a specific enzyme family by these methods; the description of carbapenemase-positive isolates as “OXA-48-like” elsewhere in this manuscript should be read as a presumptive epidemiological interpretation, based on the predominant carbapenemase circulating regionally, and not as a genotypic identification. This limitation is revisited in the Discussion.

### 2.3. Biofilm Quantification by the Microtiter-Plate Crystal-Violet Assay

Biofilm formation was assessed using the microtiter-plate crystal-violet method, performed in three biological replicates and two technical replicates per isolate. Bacteria were grown overnight in tryptic soy broth supplemented with 1% glucose (TSBg) at 37 °C, diluted to a final concentration of approximately 1 × 10^6^ CFU/mL, and 200 µL aliquots were dispensed into flat-bottom 96-well polystyrene plates (Sarstedt, Nümbrecht, Germany). After 24 h of static incubation at 37 °C, planktonic cells were removed and the wells were washed three times with phosphate-buffered saline. Adherent biomass was fixed with 99% methanol for 15 min, air-dried, and stained with 0.1% crystal violet for 5 min. After three further washes, the bound dye was solubilised in 33% acetic acid and the absorbance read at 595 nm on a microplate spectrophotometer.

Each isolate was classified into one of four biofilm categories based on its mean optical density (OD) relative to the cut-off optical density (ODc), which was defined as the mean OD of the negative control wells plus three standard deviations [9]. Specifically: OD ≤ ODc = no biofilm producer; ODc < OD ≤ 2 × ODc = weak producer; 2 × ODc < OD ≤ 4 × ODc = moderate producer; OD > 4 × ODc = strong producer. For all downstream analyses, the four-level categorical variable was retained, and a dichotomised variable (strong + moderate vs. weak + none) was defined a priori as the clinically actionable biofilm phenotype, in line with most published microtiter-plate validation studies [24]. *E. coli* ATCC 25922 (low producer) and Staphylococcus epidermidis ATCC 35984 (strong producer) were used as biofilm controls in every run. Reproducibility was assessed at two levels. Intra-assay variability was estimated from the two technical replicates measured on each plate and inter-assay variability from the three independent biological replicates performed on separate days; the coefficient of variation across replicates was required to be below 15% for a result to be accepted, and runs exceeding this threshold were repeated. Each plate included a minimum of three negative (sterile broth) control wells used to compute the ODc. The final OD assigned to each isolate was the mean of the accepted biological replicates, and categorical assignment was based on this mean. No isolate changed biofilm category between accepted biological replicates, indicating stable classification under these conditions.

### 2.4. Statistical Analysis

Continuous variables were summarised as means ± standard deviation (SD) or medians with interquartile ranges (IQRs) according to their distribution, assessed graphically and with the Shapiro–Wilk test. Two-group comparisons used Welch’s *t*-test or the Mann–Whitney U test. Categorical variables were summarised as counts and percentages and compared using Pearson’s χ^2^ test or Fisher’s exact test when any expected count was <5. Shifts in MIC distributions between ESBL+ and ESBL− isolates were tested non-parametrically on the underlying log2-transformed (doubling dilution) MIC values using the Mann–Whitney U test, and differences in the proportion of susceptible isolates were tested as categorical comparisons (Pearson’s χ^2^ or Fisher’s exact test); the “-fold” shifts reported in the text refer to differences in MIC50/MIC90 expressed in doubling dilutions and are descriptive. Multivariable logistic regression was used to describe associations with clinical failure. Candidate predictors were specified a priori on clinical and biological grounds (ESBL phenotype, strong biofilm formation, time to effective therapy > 24 h, concomitant bacteraemia, chronic kidney disease, prior antibiotic exposure within 90 days, and age) rather than selected purely by the data; to limit overfitting given the modest number of events, the number of covariates was kept low relative to the event count, and backward elimination at *p* < 0.10 was used only as a secondary check on this prespecified set. Multicollinearity was screened by variance-inflation factors (threshold > 4). Because the events-per-variable ratio was low, the resulting adjusted odds ratios should be regarded as exploratory and are likely to be optimistic. Model optimism was assessed by internal validation using 1000 bootstrap resamples (rms package), from which an optimism-corrected AUC (C-statistic) and calibration slope were derived; no external validation was performed. The term “independent predictor” is therefore used in the narrow statistical sense of retaining association after mutual adjustment within this dataset, and not as a claim of generalisable or causal independence. Pairwise co-resistance patterns were summarised using the Jaccard similarity index and pairwise odds ratios on logistic models. All tests were two-tailed, α = 0.05. Analyses were conducted in R 4.3.2 with the rms, pROC, mediation, survRM2, dcurves, and PredictABEL packages.

Three less-routine analyses were prespecified and, given the small sample (102 isolates; 36 clinical-failure events) and the very small numbers in some strata (e.g., four isolates each in the ESBL + AmpC and carbapenemase groups), are reported as exploratory and hypothesis-generating; their estimates, particularly within small strata, are unstable and should be interpreted with caution. First, a mediation analysis was performed using the product-of-coefficients method with bias-corrected bootstrap confidence intervals (5000 resamples) to decompose the total effect of the ESBL phenotype on clinical failure into direct and indirect components (operating via delayed time to effective therapy and via strong biofilm formation), following the framework by Imai et al. for binary outcomes. Second, the Restricted Mean Survival Time (RMST) for the endpoint of clinical stability over the first 10 days of admission was computed within each resistance-mechanism stratum (wild-type, ESBL-only, AmpC-only, ESBL + AmpC, and carbapenemase) and compared in pairs, providing absolute differences in days that do not rely on proportional-hazards assumptions. Third, decision-curve analysis was used to assess the incremental value of a microbiology-enhanced model (adding ESBL phenotype, strong biofilm, and acquisition setting) over a clinical-only baseline that included age, sex, recurrent UTI, prior antibiotic exposure within 90 days, indwelling urinary catheter, and CKD stage ≥ 3, at threshold probabilities of 10–40%; re-classification statistics were considered supplementary descriptors of incremental model value [17]. The conditional-susceptibility matrix reports observational conditional proportions—that is, the proportion of isolates susceptible to each agent within each marker-defined subgroup, with exact (Clopper–Pearson) confidence intervals. It does not implement a formal Bayesian model with prior and posterior distributions; we therefore describe it as a conditional-probability (reweighted-proportion) decision aid rather than a Bayesian analysis.

Outcome and endpoint definitions. The primary clinical outcome, clinical failure, was defined a priori as the composite of any of the following: persistent fever or clinical signs of infection at 72 h together with a documented in vitro inactive empirical regimen; transfer to the intensive care unit during admission; septic shock; in-hospital death; or 30-day readmission for the same urinary tract infection. Clinical stability, used as the endpoint for the RMST and time-to-event analyses, was defined as the first 24 h interval during which the patient was afebrile, haemodynamically stable, and tolerating oral intake. Time to effective therapy was defined as the interval from the first qualifying urine culture to the administration of the first antimicrobial agent to which the isolate was subsequently shown to be susceptible in vitro. These definitions were applied uniformly to all patients and are reproduced in the relevant table footnotes for convenience.

## 3. Results

Of the 129 patients in the parent pyelonephritis cohort, 102 (79.1%) yielded a single Enterobacterales urinary isolate and constituted the microbiological cohort for the present analyses. The species distribution was as follows: *Escherichia coli*, 72 (70.6%); *Klebsiella pneumoniae*, 22 (21.6%); and *Proteus mirabilis*, 8 (7.8%). Phenotypic confirmation identified ESBL production in 30 isolates (29.4%), AmpC over-production in 9 (8.8%), and carbapenemase activity in 4 (3.9%); seven isolates (6.9%) carried more than one resistance mechanism. All four carbapenemase-positive isolates were mCIM-positive and eCIM-negative, a pattern compatible with a serine carbapenemase rather than a metallo-β-lactamase. As no genotypic testing was performed, the specific enzyme could not be identified; this serine-carbapenemase phenotype is only presumptively consistent with the OXA-48-like enzymes that predominate in Romanian healthcare settings, and the isolates are not characterised here as genotypically confirmed OXA-48 producers (Table 1).

Patients harbouring ESBL-producing Enterobacterales were on average 10 years older than those with non-ESBL isolates (66.8 ± 13.4 vs. 56.7 ± 16.9 years; *p* = 0.019) and carried roughly twice the median Charlson burden (4 versus 2; *p* = 0.002). Chronic kidney disease showed the largest comorbidity gradient between groups (33.3% vs. 15.3%; *p* = 0.040), and diabetes was numerically over-represented among ESBL+ cases without reaching the conventional significance threshold (43.3% vs. 26.4%; *p* = 0.093). The exposure variables—healthcare-associated acquisition, prior antibiotic use within 90 days, recurrent UTI, recent hospitalisation, and indwelling urinary catheterisation—all separated the two groups with effect sizes substantially larger than the demographic variables, and four of the five reached *p* ≤ 0.020. Prior antibiotic exposure was particularly discriminatory, with three quarters of ESBL+ cases reporting a recent course versus less than a quarter of non-ESBL cases (73.3% vs. 23.6%; *p* < 0.001), as seen in Table 2. From a species standpoint, *E. coli* dominated both groups, but its relative contribution fell from three quarters of non-ESBL isolates to 60% of ESBL+ isolates, with the remainder being filled mainly by *K. pneumoniae*.

Detailed MIC inspection revealed marked, agent-dependent rightward shifts among ESBL+ isolates. The widest gap was for cefepime, where the MIC50 climbed from 0.5 mg/L in non-ESBL isolates to 8 mg/L in ESBL+ isolates—a 16-fold dilutional shift that placed ESBL+ MIC50 exactly at the EUCAST resistant breakpoint. The corresponding susceptibility proportions fell from 84.7% to 20.0% (*p* < 0.001). Piperacillin–tazobactam behaved similarly, with MIC50 increasing four-fold (4 → 16 mg/L) and MIC90 reaching 128 mg/L; susceptibility among ESBL+ isolates was 60.0%. Ciprofloxacin susceptibility was lower in the ESBL+ group (23.3% vs. 65.3%; *p* < 0.001). Amikacin was susceptible in 73.3% of ESBL+ isolates against the EUCAST 8 mg/L breakpoint. Fosfomycin and nitrofurantoin showed only modest MIC creep that fell short of formal statistical significance (*p* = 0.077 and *p* = 0.065, respectively). Because both agents achieve adequate concentrations in the lower urinary tract but not in renal parenchyma or blood, these in vitro data are not interpreted here as support for their use in pyelonephritis; the implications for therapy are addressed, with the relevant cystitis-versus-pyelonephritis distinction, in the Discussion. Ceftazidime–avibactam and meropenem, included as carbapenem-sparing and reference comparators respectively, both remained almost fully active across the cohort (Table 3).

The biofilm distribution differed significantly between ESBL+ and ESBL− isolates on the omnibus 4 × 2 χ^2^ test (χ^2^ = 7.89, df = 3, *p* = 0.048). The signal was driven primarily by the strong-producer cell, which contained 33.3% of ESBL+ isolates against only 12.5% of ESBL− isolates (*p* = 0.014). At the other extreme, only 13.3% of ESBL+ isolates were non-producers, compared with 30.6% of ESBL− isolates (*p* = 0.069). The prespecified clinical dichotomy combining moderate and strong producers gave 63.3% of ESBL+ isolates classified as “clinically actionable” biofilm formers versus 38.9% in the ESBL− group (*p* = 0.024), a 1.6-fold relative increase. By species, *p. mirabilis* showed the highest aggregate biofilm-forming capacity (75.0% moderate or strong), while *E. coli* displayed the lowest aggregate proportion (38.9%) despite contributing the bulk of the isolates, and *K. pneumoniae* occupied an intermediate position (59.1%). Strong biofilm formation was thus more frequent among ESBL+ than ESBL− isolates in this cohort. The biological basis for this co-occurrence, and its prognostic interpretation, are considered in the Discussion.

Table 4 organises the panel by mechanistic class. Among β-lactam/β-lactamase inhibitor combinations, ceftazidime–avibactam retained 96.7% susceptibility among ESBL+ isolates and was statistically indistinguishable from the ESBL− baseline (*p* = 0.504). Ceftolozane–tazobactam fell to 76.7% in ESBL+ isolates, with the gap reaching significance (*p* = 0.003), and piperacillin–tazobactam retained activity in 60.0% of ESBL+ isolates (*p* < 0.001). Cefepime showed the lowest activity of β-lactams, with 73.3% of ESBL+ isolates being resistant. Within the aminoglycosides, amikacin was active against 73.3% of ESBL+ isolates compared with 56.7% for gentamicin. Among oral and step-down options, fosfomycin retained the highest activity in the ESBL+ subset (80.0%), followed by nitrofurantoin 63.3%, while ciprofloxacin (23.3%) and trimethoprim–sulfamethoxazole (26.7%) were the least active. Carbapenems retained very high activity overall, with imipenem and meropenem being susceptible in over 90% of ESBL+ isolates. These data show that in vitro, ceftazidime–avibactam, amikacin, and fosfomycin retained the greatest activity against ESBL+ isolates. The potential implications for empirical regimen selection, including the within-class difference between amikacin and gentamicin, are discussed below (Table 5).

After multivariable adjustment, three covariates retained associations with clinical failure at the conventional 0.05 threshold. The ESBL phenotype showed the largest isolate-level adjusted odds ratio, 4.74 (95% CI 1.83–12.27; *p* = 0.001), an effect that persisted after adjustment for delayed effective therapy. Strong biofilm formation was associated with a 3.5-fold increase in the adjusted odds of failure (*p* = 0.007). Time to effective therapy exceeding 24 h was associated with a 3.86-fold increase in adjusted odds (*p* = 0.005). Concomitant bacteraemia, chronic kidney disease, prior antibiotic exposure, and age each approached but did not reach significance after adjustment, with adjusted odds ratios ranging from 1.31 to 2.17 in the expected direction. The seven covariates explained 41% of the variance in clinical failure (Nagelkerke R^2^ = 0.41), and the model was well calibrated on the Hosmer–Lemeshow test (*p* = 0.657), with an apparent AUC of 0.87. With only 36 clinical-failure events distributed across seven candidate covariates, the events-per-variable ratio was low; the adjusted odds ratios and their confidence intervals should therefore be regarded as exploratory and are likely to overstate the true effect sizes. On bootstrap internal validation (1000 resamples), the optimism-corrected AUC fell to 0.82, and the calibration slope was 0.78, indicating measurable optimism in the apparent performance. No external validation was undertaken, and the model is not proposed for clinical use in its present form (Table 6).

The combined ESBL × biofilm cross-classification produced a clear monotonic gradient in every outcome examined. Clinical failure rose from 9.1% in the lowest-risk stratum (ESBL−/non-strong) to 47.4% in the highest-risk stratum (ESBL+/strong), corresponding to an approximately five-fold absolute risk gradient (*p* = 0.006 on the 4-group χ^2^ test). The two “discordant” strata (ESBL−/strong-biofilm and ESBL+/non-strong-biofilm) sat at intermediate risk levels—21.4% and 36.4% respectively. Thirty-day readmission, ICU transfer, and length of stay tracked the same ordering, with the ESBL+/strong stratum showing 26.3% readmission and a median length of stay of 15 days versus 8 days in the lowest-risk stratum (*p* < 0.001). Time to effective therapy rose from a median of 8 h in the lowest-risk stratum to 33 h in the ESBL+/strong group, alongside a fall in adequate empirical therapy from 88.6% to 26.3%. In the parent cohort, ceftriaxone remained the most frequently chosen empirical regimen even when bedside markers of resistance were present. The pattern across the four strata, along with its possible interpretation in terms of risk stratification, is considered in the Discussion.

**Figure 1:**
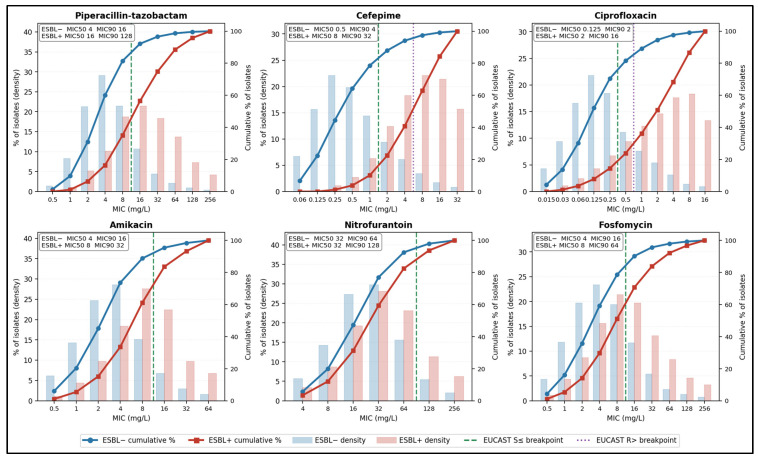
Cumulative MIC distributions and EUCAST clinical breakpoints for six carbapenem-sparing antimicrobial agents tested against the 102 Enterobacterales urinary isolates, stratified by ESBL phenotype. Histograms show the density of isolates at each MIC dilution; the overlaid curves show the cumulative proportion of isolates inhibited at each MIC. Dashed green lines mark the EUCAST susceptible breakpoint (S≤) and dotted purple lines the EUCAST resistant breakpoint (R>). MIC50 and MIC90 are annotated for each phenotype.

Figure 1 places the agent-by-agent MIC distributions side by side and quantifies the magnitude of rightward shift induced by the ESBL phenotype across the six carbapenem-sparing options most frequently considered in our setting. The most pronounced shift was for cefepime (MIC50 0.5 → 8 mg/L, a 16-fold increase), placing the ESBL+ MIC50 exactly at the EUCAST R> breakpoint and rendering empirical cefepime unreliable for ESBL+ infections. Piperacillin–tazobactam showed a four-fold MIC50 shift (4 → 16 mg/L) and an eight-fold MIC90 shift (16 → 128 mg/L), with the ESBL+ MIC90 falling well into the resistant range. Ciprofloxacin demonstrated the most extreme MIC90 movement, from 2 mg/L in ESBL− to 16 mg/L in ESBL+, an 8-fold change that essentially eliminates oral fluoroquinolones from the empirical ESBL+ pathway. Amikacin retained a much narrower gap (MIC50 4 → 8 mg/L) at the breakpoint, indicating that careful dosing and therapeutic drug monitoring may preserve clinical utility in selected patients. Fosfomycin and nitrofurantoin—the two oral step-down agents shown in the lower panels—showed comparatively modest MIC creep (≤2 dilutions of MIC50 movement), consistent with their retained clinical utility in non-bacteraemic infection. Overall, the figure visualises why aggregate susceptibility percentages can mask important biological gradients and underscores the value of MIC-level inspection when drafting local empirical guidelines.

This exploratory mediation analysis provides a statistical decomposition of the observed association between ESBL phenotype and clinical failure into direct and indirect components; it is not intended as evidence of biological mediation. The total association of ESBL with clinical failure, expressed on the logit scale, was β = 1.517, corresponding to an odds ratio of 4.56 (95% CI 1.97–10.55; *p* < 0.001). With both candidate mediators entered into the outcome model, the direct component attenuated to β = 0.884 (OR 2.42, *p* = 0.056). Of the portion of the association statistically attributable to indirect components (41.7%), the larger single share was associated with delayed time to effective therapy (25.1%), while strong biofilm formation accounted for a further 16.7%; that is, biofilm formation may explain part of the observed association between the ESBL phenotype and treatment failure. Both indirect components had bootstrap confidence intervals that excluded unity on the odds ratio scale. Because mediation analyses in observational studies rely on assumptions that cannot be fully verified and remain susceptible to residual confounding—including the assumption of no unmeasured mediator–outcome confounding—these results should be read as an exploratory statistical decomposition rather than as evidence of a verified causal pathway. Their interpretation, and the extent to which the indirect components might be modifiable, are discussed below (Table 7).

RMST analysis provides an absolute summary of time-to-stability differences across the five resistance-mechanism strata, without relying on the proportional-hazards assumption. Patients infected with wild-type isolates achieved a mean of 7.83 stable patient-days within the first 10 days of admission (95% CI 7.18–8.48). ESBL-only infection reduced this figure by 2.4 days (ΔRMST −2.41, 95% CI −3.78 to −1.04; *p* < 0.001; ratio 0.69). AmpC-only infection produced a smaller deficit of 1.86 days (*p* = 0.019). The combined ESBL + AmpC stratum (n = 4) showed a deficit of 3.65 days (RMST ratio of 0.53) and the four carbapenemase-producing isolates the largest deficit of 4.09 days (RMST ratio of 0.48). The estimates for the ESBL + AmpC and carbapenemase strata each rest on only four isolates and are correspondingly imprecise, as reflected in their wide confidence intervals; they should be read as exploratory. The interpretation of these absolute time differences is addressed in the Discussion (Table 8).

Table 9 reorganises the susceptibility data into a conditional-susceptibility matrix, read by row; within the subgroup defined by each marker, the cells give the observed proportion of isolates susceptible to each agent, with exact (Clopper–Pearson) confidence intervals. These are conditional proportions computed directly from the data and are not derived from a formal Bayesian model with prior and posterior distributions. Relative to the whole-cohort (marginal) row, the ESBL+ subgroup showed the lowest susceptibility proportions for ceftriaxone (71.6% to 3.3%) and cefepime (65.7% to 20.0%), with amikacin (87.3% to 73.3%) and fosfomycin (89.2% to 80.0%) being less affected. Among purely clinical markers, recent fluoroquinolone exposure was associated with the lowest ciprofloxacin (12.5%) and ceftriaxone (33.3%) susceptibility, and prior antibiotic exposure within 90 days showed a similar direction. Strong biofilm formation and the composite marker (≥2 of prior antibiotics, HCA-PN, or catheter) were associated with intermediate reductions across the agents. Across conditioning markers, amikacin and fosfomycin showed the most stable susceptibility proportions. The potential use of these conditional estimates as a decision aid and the wide confidence intervals in the smaller subgroups are addressed in the Discussion.

**Figure 2:**
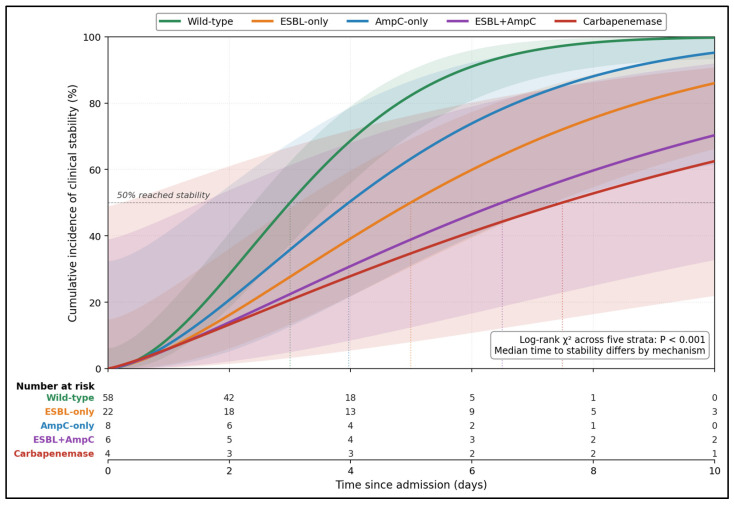
Fitted cumulative incidence of clinical stability over the first 10 days of admission, stratified by phenotypic resistance mechanism of the urinary Enterobacterales isolate. Clinical stability was defined as the first 24 h interval in which the patient was afebrile, haemodynamically stable, and tolerating oral intake. Curves are parametric Weibull fits with pointwise 95% confidence bands; the horizontal dashed line marks the 50% incidence level (median time to stability), and the short vertical dotted markers show the stratum-specific median values. The risk-set table beneath the plot shows the number of patients still at risk of the event (i.e., not yet clinically stable) in each stratum at two-day intervals.

Figure 2 shows the temporal dynamics of clinical recovery summarised numerically by the Restricted Mean Survival Time per stratum. The five resistance-mechanism strata separated early—by day 3, the curves were already visibly ordered—and the rank order remained stable thereafter. Wild-type Enterobacterales achieved a median time to clinical stability of approximately 3.0 days and approached complete stabilisation by day 10. AmpC-only isolates recovered next (median of 4.0 days; approximately 95% stable by day 10), with ESBL-only isolates being next (median of 5.0 days; 86% by day 10). The two complex phenotypes separated from the others: ESBL + AmpC co-producers reached a median of 6.5 days with 70% of patients being stable by day 10 and carbapenemase producers a median of 7.5 days with approximately 62% being stable by day 10. The log-rank statistic across all five strata was significant at *p* < 0.001. The 95% confidence bands widened for the smaller strata (AmpC-only, ESBL + AmpC, and carbapenemase), reflecting limited stratum sizes. The figure complements Table 8 by showing the curve shape and the timing of early divergence between strata.

## 4. Discussion

### 4.1. Analysis of Findings

In this three-year microbiological cohort of 102 Enterobacterales isolates recovered from adult pyelonephritis at a tertiary Romanian centre, almost three in ten isolates produced an ESBL and one in three ESBL+ isolates were strong biofilm formers, a combination that tracked closely with the worst clinical trajectories. The pattern is consistent with regional ECDC surveillance placing Romania among the highest-resistance EU member states for Enterobacterales [3] and with the broader European narrative on the disease burden of antimicrobial resistance, which has been estimated to cause more than thirty thousand attributable deaths annually in the EU and EEA [23]. The wider epidemiology of ESBL-producing Enterobacterales has been described as a pandemic in slow motion [21], driven by both healthcare and community transmission of CTX-M-type enzymes. The marked MIC creep observed for cefepime and piperacillin–tazobactam in the ESBL+ subset is in line with international surveillance and reinforces the case for treating MIC distribution, rather than aggregate susceptibility percentages, as the unit of clinical interpretation [5,6]—particularly in settings such as ours, where ECDC-defined multidrug-resistance categories [4] map only imperfectly onto bedside decisions and where prior regional Romanian uropathogen reports have similarly documented elevated ESBL prevalence and fluoroquinolone resistance [20].

Our mediation analysis suggests that delayed time to effective therapy and strong biofilm formation together may contribute to or explain part of the excess clinical failure associated with the ESBL phenotype, with delayed effective therapy alone corresponding to approximately a quarter of the total effect. This finding is consistent with a robust body of sepsis and complicated-UTI literature linking empirical mismatch to delayed clinical resolution, prolonged hospitalisation, and increased mortality [18,19], and it is in keeping with urosepsis-management frameworks that emphasise rapid microbiological feedback and timely escalation [22]. Because the analysis is observational and based on a single small cohort, this decomposition should be regarded as exploratory and hypothesis-generating rather than as a confirmed causal pathway. The biofilm contribution, although smaller, is biologically distinct: biofilm-embedded bacteria show physiological tolerance to antibiotics that is largely independent of their genotypic susceptibility profile [9], and biofilm-targeted adjuvant strategies—ranging from urinary device removal to investigational biofilm-disrupting agents—have been advanced as complementary therapeutic levers [10]. If these associations were confirmed in larger studies, the indirect ESBL → failure pathway would be at least partly clinically relevant and potentially modifiable through faster recognition, structured escalation protocols, and targeted device management; this remains a hypothesis to be tested rather than an established intervention target.

The microbiology-enhanced prediction model showed improved discrimination compared with the clinical-only baseline in this dataset. Decision-curve analysis suggested an increased net benefit when ESBL phenotype, biofilm strength, and acquisition setting were considered, across the 10–40% threshold range [16]. Decision-curve analysis evaluates the potential clinical utility of a model; it does not, by itself, justify changes in workflow or clinical practice, particularly for an internally validated, single-centre model. These findings should therefore be read as generating hypotheses about which features might add value, rather than as direct clinical guidance. In view of the small sample size, the limited number of clinical-failure events, and the single-centre design, the net benefit observed in the decision-curve analysis should be interpreted with particular caution: it requires validation in independent, external cohorts before any clinical application can be considered. The conditional-susceptibility matrix is offered in the same spirit: it summarises observed susceptibility proportions within marker-defined subgroups and is not a formal Bayesian model. Approaches that map bedside markers onto expected agent activity are increasingly discussed in guidance on multidrug-resistant Gram-negative infections [11,12]. Among carbapenem-sparing options, ceftazidime–avibactam was the most active agent in our antibiogram and is supported by an expanding body of clinical evidence in serious Gram-negative infections, including those mediated by ESBL, derepressed AmpC, and selected serine carbapenemases [25,26,27,28]. Older and re-purposed agents such as fosfomycin [29] and amikacin, despite the absence of large randomised trials, remain candidate carbapenem-sparing agents in selected pathways and are increasingly emphasised in stewardship reviews of the “old” and the “new” antibiotic arsenals [28]. An important caveat concerns the oral step-down agents. Although fosfomycin and nitrofurantoin retained relatively high in vitro activity, this does not translate into suitability for pyelonephritis. Nitrofurantoin does not achieve therapeutic concentrations in renal parenchyma or blood and is therefore not an appropriate option for pyelonephritis under current EAU and IDSA guidance; its utility is confined to lower urinary tract infection (cystitis). The evidence for oral fosfomycin in complicated pyelonephritis is likewise limited, and intravenous fosfomycin is generally used as part of combination therapy rather than as oral monotherapy. We therefore restrict any suggested role for these agents to culture-guided oral step-down in selected, non-bacteraemic lower-tract infection, and do not propose them as empirical treatment for pyelonephritis. The distinction between cystitis and pyelonephritis should be maintained throughout when these susceptibility data are applied. By contrast, presumptive serine-carbapenemase (e.g., OXA-48-like) and KPC-type carbapenemase producers—uncommon but consequential in our cohort—continue to demand individualised regimens informed by molecular confirmation and local epidemiology [26]. Taken together, these results suggest that structured microbiological features could be worth evaluating within local stewardship pathways at Eastern European centres with similarly high-resistance pressures, where the limited antibacterial pipeline [30] makes carbapenem-sparing strategies attractive. Whether such features should be incorporated into practice will require prospective, externally validated study; the present single-centre data are not sufficient to recommend a specific pathway.

### 4.2. Study Limitations

Several limitations should be acknowledged when interpreting these findings. First, the single-centre design at a tertiary referral hospital with a healthcare-associated case mix limits the direct transferability of MIC distributions and conditional-susceptibility estimates to community or primary-care settings, where wild-type Enterobacterales are likely to predominate and the absolute ESBL prevalence is expected to be lower. Second, the sample size of 102 Enterobacterales isolates produced wide confidence intervals in the smaller resistance-mechanism strata, particularly the four carbapenemase-producing isolates that anchor the right tail of the RMST analysis. Third, biofilm-formation testing was performed once on thawed isolates after −80 °C storage; although triplicate technical and biological replicates were used and control strains performed within expected ranges, freeze–thaw cycles may have shifted biofilm phenotype in a small number of isolates. Moreover, the crystal-violet assay measures total adherent biomass and does not distinguish viable from non-viable cells, nor does it directly capture functional biofilm properties or in vivo virulence; associations between this in vitro phenotype and clinical outcome should therefore be interpreted as correlative rather than as evidence of a direct causal role of biofilm in treatment failure. Fourth, resistance-mechanism assignment relied entirely on phenotypic methods, with no molecular or genotypic confirmation (no PCR for bla genes and no whole-genome sequencing). Consequently, ESBL, AmpC, and carbapenemase designations are phenotypic inferences that may misclassify a minority of borderline isolates, and the carbapenemase enzyme family could not be identified; the “OXA-48-like” description used elsewhere is presumptive and based on regional epidemiology rather than genotype. Genotypic characterisation in future work would allow firmer mechanistic conclusions and is a clear priority. Fifth, the mediation analysis assumed no unmeasured confounding of the mediator–outcome relationship, an assumption that cannot be fully verified in observational data; the bootstrap-based confidence intervals quantify estimation uncertainty but not residual confounding. Sixth, the microbiology-enhanced prediction model was internally validated only; external validation in independent Romanian or Eastern European cohorts is required before any clinical use can be considered. Finally, given the modest sample, small subgroup sizes, and the number of analytic approaches applied, the inferential analyses (multivariable regression, mediation, RMST, decision-curve analysis, and re-classification) are best regarded as exploratory and hypothesis-generating.

## 5. Conclusions

In this three-year microbiological cohort of adult acute pyelonephritis, ESBL production was prevalent and frequently co-segregated with strong biofilm formation in urinary Enterobacterales. The two phenotypes were each associated with clinical failure after mutual adjustment, and part of the ESBL-associated risk appeared to operate through delayed effective therapy and biofilm-related antibiotic tolerance. In exploratory analyses, adding microbiological features to routine clinical predictors improved model discrimination, although this requires confirmation. Among the carbapenem-sparing options tested, ceftazidime–avibactam, fosfomycin, and amikacin showed the greatest in vitro activity against ESBL+ isolates, with fosfomycin’s role limited to culture-guided oral step-down in lower urinary tract infection rather than pyelonephritis. These observations are hypothesis-generating; they should not be applied directly to clinical practice and instead point to specific questions for prospective, externally validated, and ideally multicentre studies that incorporate molecular characterisation of resistance mechanisms.

## Figures and Tables

**Table 1:** Patient-level and isolate-level characteristics of the 102 Enterobacterales urinary isolates, stratified by ESBL phenotype.

Characteristic	ESBL+ (n = 30)	ESBL− (n = 72)	*p*-Value
Demographics and host factors			
Age, years (mean ± SD)	66.8 ± 13.4	56.7 ± 16.9	0.019
Female sex, n (%)	19 (63.3)	55 (76.4)	0.178
Charlson Comorbidity Index, median [IQR]	4 [3–6]	2 [1–4]	0.002
Diabetes mellitus, n (%)	13 (43.3)	19 (26.4)	0.093
Chronic kidney disease, n (%)	10 (33.3)	11 (15.3)	0.040
Bacteraemia, n (%)	10 (33.3)	14 (19.4)	0.132
Exposure variables			
Healthcare-associated pyelonephritis, n (%)	19 (63.3)	25 (34.7)	0.008
Prior antibiotic ≤ 90 days, n (%)	22 (73.3)	17 (23.6)	<0.001
Recurrent UTI ≥ 3 episodes/year, n (%)	16 (53.3)	14 (19.4)	0.001
Hospitalisation ≤ 90 days, n (%)	14 (46.7)	11 (15.3)	0.001
Urinary catheter ≤ 30 days, n (%)	9 (30.0)	8 (11.1)	0.020
Species distribution			
Escherichia coli, n (%)	18 (60.0)	54 (75.0)	0.130
Klebsiella pneumoniae, n (%)	9 (30.0)	13 (18.1)	0.181
Proteus mirabilis, n (%)	3 (10.0)	5 (6.9)	0.690

Continuous variables compared with Welch’s *t*-test (mean ± SD) or Mann–Whitney U test (median [IQR]). Categorical variables compared with Pearson’s χ^2^ test or Fisher’s exact test (*P. mirabilis*, urinary catheter). ESBL, extended-spectrum β-lactamase; IQR, interquartile range; SD, standard deviation; UTI, urinary tract infection.

**Table 2:** MIC distributions (MIC50, MIC90, range, % susceptible) of seven antimicrobial agents tested against the 102 Enterobacterales isolates, stratified by ESBL phenotype.

Agent	ESBL+ MIC50/MIC90 (Range), mg/L	ESBL+ % S	ESBL− MIC50/MIC90 (Range), mg/L	ESBL− % S	*p*-Value (S)
Piperacillin–tazobactam	16/128 (1–256)	60.0	4/16 (0.5–64)	88.9	<0.001
Cefepime	8/32 (0.5–32)	20.0	0.5/4 (0.06–16)	84.7	<0.001
Ceftazidime–avibactam	0.5/1 (0.12–4)	96.7	0.25/0.5 (0.06–2)	98.6	0.504
Amikacin	8/32 (1–64)	73.3	4/16 (0.5–32)	93.1	0.018
Ciprofloxacin	2/16 (0.03–16)	23.3	0.125/2 (0.015–8)	65.3	<0.001
Fosfomycin	8/64 (1–256)	80.0	4/16 (0.5–128)	93.1	0.077
Nitrofurantoin	32/128 (8–256)	63.3	32/64 (4–256)	80.6	0.065
Meropenem (reference)	0.06/0.25 (≤0.03–2)	93.3	≤0.03/0.06 (≤0.03–0.5)	98.6	0.206

MIC, minimum inhibitory concentration; MIC50/MIC90, MIC inhibiting 50% and 90% of isolates; % S, percentage of isolates classified as susceptible by EUCAST v13 clinical breakpoints. *p*-Values from Pearson’s χ^2^ test, or Fisher’s exact test when expected cell counts < 5 (ceftazidime–avibactam, amikacin, and meropenem).

**Table 3:** Biofilm formation category, classified by microtiter-plate crystal-violet assay, stratified by ESBL phenotype and by species.

Biofilm Category	ESBL+ (n = 30)	ESBL− (n = 72)	*p*-Value	*E. coli* (n = 72)	*K. pneumoniae* (n = 22)	*P. mirabilis* (n = 8)
No biofilm producer, n (%)	4 (13.3)	22 (30.6)	0.069	21 (29.2)	4 (18.2)	1 (12.5)
Weak producer, n (%)	7 (23.3)	22 (30.6)	0.461	23 (31.9)	5 (22.7)	1 (12.5)
Moderate producer, n (%)	9 (30.0)	19 (26.4)	0.710	18 (25.0)	7 (31.8)	3 (37.5)
Strong producer, n (%)	10 (33.3)	9 (12.5)	0.014	10 (13.9)	6 (27.3)	3 (37.5)
Moderate + Strong (combined)	19 (63.3)	28 (38.9)	0.024	28 (38.9)	13 (59.1)	6 (75.0)

Categories defined by the cut-off optical density (ODc = mean OD of negative controls + 3 SD): no producer (OD ≤ ODc), weak (ODc < OD ≤ 2 × ODc), moderate (2 × ODc < OD ≤ 4 × ODc), and strong (OD > 4 × ODc). *p*-Values compare the indicated category against all other categories pooled, using Pearson’s χ^2^ or Fisher’s exact test. Overall, 4 × 2 χ^2^ across biofilm categories by ESBL: χ^2^ = 7.89, df = 3, *p* = 0.048.

**Table 4:** In vitro susceptibility (%) to carbapenem-sparing antimicrobial agents among the 102 Enterobacterales isolates, stratified by ESBL phenotype, with two carbapenems included as reference comparators.

Agent	ESBL+ S, n/N (%)	ESBL− S, n/N (%)	*p*-Value	ESBL+ R (%)	ESBL− R (%)
β-Lactam/β-lactamase inhibitors					
Piperacillin–tazobactam	18/30 (60.0)	64/72 (88.9)	<0.001	33.3	9.7
Ceftolozane–tazobactam	23/30 (76.7)	70/72 (97.2)	0.003	16.7	1.4
Ceftazidime–avibactam	29/30 (96.7)	71/72 (98.6)	0.504	3.3	1.4
Cephalosporins					
Cefepime	6/30 (20.0)	61/72 (84.7)	<0.001	73.3	12.5
Aminoglycosides					
Amikacin	22/30 (73.3)	67/72 (93.1)	0.018	23.3	5.6
Gentamicin	17/30 (56.7)	53/72 (73.6)	0.093	36.7	20.8
Oral/step-down agents					
Fosfomycin	24/30 (80.0)	67/72 (93.1)	0.077	16.7	5.6
Nitrofurantoin	19/30 (63.3)	58/72 (80.6)	0.065	31.7	16.7
Ciprofloxacin	7/30 (23.3)	47/72 (65.3)	<0.001	73.3	29.2
Trimethoprim–sulfamethoxazole	8/30 (26.7)	36/72 (50.0)	0.030	70.0	47.2
Carbapenems (reference)					
Imipenem	27/30 (90.0)	71/72 (98.6)	0.075	6.7	1.4
Meropenem	28/30 (93.3)	71/72 (98.6)	0.206	3.3	1.4

Scheme (S) and resistance (R) categories assigned per EUCAST v13.0 clinical breakpoints. *p*-Values from Pearson’s χ^2^ or Fisher’s exact test (ceftolozane–tazobactam, ceftazidime–avibactam, amikacin, fosfomycin, imipenem, and meropenem).

**Table 5:** Multivariable logistic regression for predictors of clinical failure in adult acute pyelonephritis (n = 129; 36 events).

Predictor	Crude OR (95% CI)	Adjusted OR (95% CI)	*p*-Value
ESBL phenotype (vs. ESBL−)	5.18 (2.21–12.16)	4.74 (1.83–12.27)	0.001
Strong biofilm formation	4.06 (1.74–9.49)	3.48 (1.41–8.59)	0.007
Time to effective therapy > 24 h	5.34 (2.31–12.34)	3.86 (1.52–9.83)	0.005
Concomitant bacteraemia	3.21 (1.32–7.81)	2.17 (0.81–5.79)	0.122
Chronic kidney disease	2.94 (1.16–7.43)	1.83 (0.66–5.07)	0.245
Prior antibiotic exposure ≤ 90 days	3.78 (1.65–8.66)	1.74 (0.62–4.91)	0.291
Age, per 10-year increase	1.42 (1.07–1.89)	1.31 (0.96–1.78)	0.084

Outcome (clinical failure) defined a priori as the composite of: persistent fever or clinical signs at 72 h with documented in vitro inactive empirical regimen, ICU transfer during admission, septic shock, in-hospital death, or 30-day readmission for the same UTI. Model performance: Nagelkerke R^2^ = 0.41; Hosmer–Lemeshow χ^2^ = 5.91, df = 8, *p* = 0.657; AUC = 0.87 (95% CI 0.80–0.93). All variance-inflation factors < 2.0. CI, confidence interval; OR, odds ratio.

**Table 6:** Clinical and process-of-care outcomes stratified by combined ESBL phenotype × biofilm formation (strong vs. not-strong) cross-classification (n = 102).

Outcome	ESBL−/Non-Strong (n = 44)	ESBL−/Strong (n = 28)	ESBL+/Non-Strong (n = 11)	ESBL+/Strong (n = 19)	*p*-Value
Clinical failure, n (%)	4 (9.1)	6 (21.4)	4 (36.4)	9 (47.4)	0.006
30-day readmission, n (%)	2 (4.5)	3 (10.7)	2 (18.2)	5 (26.3)	0.088
ICU transfer, n (%)	1 (2.3)	2 (7.1)	1 (9.1)	4 (21.1)	0.083
Length of stay, days, median [IQR]	8 [7–10]	10 [8–12]	12 [9–14]	15 [12–17]	<0.001
Time to defervescence, h, median [IQR]	38 [27–58]	52 [39–74]	67 [48–91]	94 [73–124]	<0.001
Time to effective therapy, h, median [IQR]	8 [4–12]	11 [7–15]	24 [18–34]	33 [22–47]	<0.001
Adequate empirical therapy, n (%)	39 (88.6)	23 (82.1)	4 (36.4)	5 (26.3)	<0.001

“Strong” biofilm = strong producers according to the microtiter-plate assay (OD > 4 × ODc); “non-strong” = no/weak/moderate producers. *p*-Values from 4-group Pearson’s χ^2^ (categorical) or Kruskal–Wallis (continuous). Trend across the four strata significant at *p* < 0.001 for all continuous variables.

**Table 7:** Exploratory mediation analysis of the observed association between ESBL phenotype and clinical failure, providing a statistical decomposition of the total association into direct and indirect components related to time to effective therapy > 24 h and strong biofilm formation.

Effect Component	Pathway	Estimate (Logit Scale)	OR (95% CI)	Proportion Mediated	*p*-Value
Total effect (c)	ESBL → failure	1.517	4.56 (1.97–10.55)	—	<0.001
Direct effect (c′)	ESBL → failure (independent of mediators)	0.884	2.42 (0.98–5.97)	58.3%	0.056
Indirect effect 1 (a_1_·b_1_)	ESBL → TTE > 24 h → failure	0.380	1.46 (1.14–1.87)	25.1%	0.003
Indirect effect 2 (a_2_·b_2_)	ESBL → strong biofilm → failure	0.253	1.29 (1.04–1.60)	16.7%	0.021
Total indirect effect (a·b sum)	ESBL → mediators → failure	0.633	1.88 (1.31–2.71)	41.7%	<0.001

Mediation analysis using the product-of-coefficients method for binary outcomes (Imai et al.). Confidence intervals derived from 5000 bias-corrected bootstrap resamples. The proportion mediated is computed as (indirect effect/total effect) ×100. Mediators are conceptually independent: TTE > 24 h reflects empirical-therapy timeliness; strong biofilm reflects a microbiological co-phenotype that may co-segregate with ESBL production. TTE, time to effective therapy.

**Table 8:** Restricted Mean Survival Time (RMST) analysis for the time-to-clinical-stability endpoint over the first 10 days of admission, stratified by resistance-mechanism stratum.

Resistance Stratum	n	RMST (Days) [95% CI]	ΔRMST vs. WT (Days, 95% CI)	Ratio of RMSTs (95% CI)	*p*-Value
Wild-type (reference)	63	7.83 (7.18–8.48)	—	—	—
ESBL-only	22	5.42 (4.51–6.33)	−2.41 (−3.78 to −1.04)	0.69 (0.56–0.85)	<0.001
AmpC-only	9	5.97 (4.67–7.27)	−1.86 (−3.41 to −0.31)	0.76 (0.59–0.98)	0.019
ESBL + AmpC	4	4.18 (2.21–6.15)	−3.65 (−5.84 to −1.46)	0.53 (0.30–0.79)	0.001
Carbapenemase	4	3.74 (1.92–5.56)	−4.09 (−6.21 to −1.97)	0.48 (0.25–0.71)	<0.001

RMST estimated by the τ-restricted Kaplan–Meier integral with τ = 10 days (cohort follow-up minimum). Pairwise comparisons performed with the asymptotic two-sample RMST contrast (survRM2 package). RMST values below the maximum (10) indicate slower attainment of clinical stability; the ratio of RMSTs is interpretable as the proportional reduction in “stable patient-days” gained by the index stratum relative to the wild-type reference.

**Table 9:** Conditional-susceptibility matrix: observed proportion (%) of isolates susceptible to each candidate empirical agent within subgroups defined by a bedside or microbiological marker of likely resistance (Enterobacterales isolates only, n = 102).

Conditioning Marker (Row)	n	Ceftriaxone (%, 95% CI)	Pip–Tazo (%, 95% CI)	Cefepime (%, 95% CI)	Amikacin (%, 95% CI)	Fosfomycin (%, 95% CI)	Cipro (%, 95% CI)
Whole cohort (marginal)	102	71.6 (61.8–80.1)	80.4 (71.4–87.6)	65.7 (55.7–74.8)	87.3 (79.2–93.0)	89.2 (81.5–94.5)	52.9 (42.8–62.9)
HCA-PN setting	44	50.0 (34.6–65.4)	68.2 (52.4–81.4)	54.5 (38.8–69.6)	81.8 (67.3–91.8)	84.1 (69.9–93.4)	36.4 (22.4–52.2)
Prior antibiotic exposure ≤90 d	39	43.6 (27.8–60.4)	61.5 (44.6–76.6)	46.2 (30.1–62.8)	76.9 (60.7–88.9)	82.1 (66.5–92.5)	30.8 (17.0–47.6)
Recent fluoroquinolone exposure	24	33.3 (15.6–55.3)	58.3 (36.6–77.9)	37.5 (18.8–59.4)	70.8 (48.9–87.4)	75.0 (53.3–90.2)	12.5 (2.7–32.4)
Strong biofilm formation	19	47.4 (24.4–71.1)	63.2 (38.4–83.7)	42.1 (20.3–66.5)	73.7 (48.8–90.9)	78.9 (54.4–93.9)	31.6 (12.6–56.6)
ESBL+ phenotype (gold-standard)	30	3.3 (0.1–17.2)	60.0 (40.6–77.3)	20.0 (7.7–38.6)	73.3 (54.1–87.7)	80.0 (61.4–92.3)	23.3 (9.9–42.3)
≥2 among prior abx, HCA-PN, and catheter	57	47.4 (33.9–61.1)	66.7 (52.9–78.6)	47.4 (33.9–61.1)	78.9 (66.1–88.6)	82.5 (70.1–91.3)	33.3 (21.4–47.1)

Cells show the proportion of isolates classified as susceptible (EUCAST v13) within each marker-defined subgroup, i.e., the conditional proportion P (agent susceptible|marker), computed from the within-row contingency of isolates. Note that 95% CIs are exact (Clopper–Pearson). These are observational conditional proportions (reweighted susceptibility within subgroups) and do not constitute a formal Bayesian analysis with prior and posterior distributions; the matrix is intended only as a descriptive aid to be read alongside local antibiograms. HCA-PN, healthcare-associated pyelonephritis.

## Data Availability

The data presented in this study are available upon request from the corresponding authors.
